# Laboratory-Scale Evaluation of a Plant-Based Algaecide for Harmful and Non-Harmful Algae

**DOI:** 10.3390/toxins17060270

**Published:** 2025-05-27

**Authors:** Raphael M. Kudela

**Affiliations:** Institute of Marine Sciences, University of California Santa Cruz, Santa Cruz, CA 95064, USA; kudela@ucsc.edu

**Keywords:** harmful algal blooms, treatment, *Microcystis*, microcystin, *Pseudo-nitzschia*, *Chattonella*, *Heterosigma akashiwo*

## Abstract

Harmful algal blooms can negatively impact freshwater, estuarine, and coastal marine systems globally and pose serious risks to water quality, human and ecosystem health, and food production. Algae can produce toxic compounds, directly interfere with aquaculture species through (e.g.,) the production of foam or mucilage, as well as causing diseases and disorders in fish, and can result in hypoxic conditions when the bloom senesces. Application of US Environmental Protection Agency (USEPA) registered algaecides can be effective, scalable, and inexpensive, but there is growing interest in plant- or bacterial-derived compounds that do not require the use of chemicals such as hydrogen peroxide or copper. The algaecide C7X1 is a plant-based organic algaecide that proves effective against a wide variety of algae, including harmful algal species such as *Microcystis*, *Heterosigma*, and *Pseudo-nitzschia*. Performance is comparable to other USEPA-registered algaecides, with low to moderate extracellular toxin release and a potential lifetime of weeks in treated waters. The mode of action is inhibition of photosynthesis, suggesting that direct off-target impacts on zooplankton and other organisms would be minimal.

## 1. Introduction

Harmful algal blooms (HABs) are ubiquitous in freshwater, brackish, and marine environments [1,2,3,4]. HABs can include microalgae, cyanobacteria, and macroalgae and can include both low and high biomass events. Many HABs are also toxic, including *Pseudo-nitzschia*, which produces domoic acid [5], *Microcystis*, which produces microcystins [6], *Heterosigma akashiwo* and *Chattonella*, which produce reactive oxygen species [7,8], and *Anabaena flos-aquae*, which can produce anatoxins [9]. Freshwater HAB toxins are finding their way into marine environments and contaminating seafood with poorly understood consequences [10,11,12,13]. Blooms of fish and shellfish killing HABs are occurring in many regions and are especially threatening to aquaculture [14]. While long-term preventive mitigation and management efforts (i.e., nutrient reduction) are optimal [15,16], the problem is formidable, leading to both costly and long-term (years to decades) solutions to address these issues. Blooms threatening public and environmental health may be best mitigated through short-term solutions, including immediate control, such as the use of USEPA-registered chemical algaecides (c.f. [17]). However, use of commercially available algaecides can be problematic. Some use copper as an inhibitor [18], many others use oxidants such as hydrogen peroxide [19], and there are numerous regions that restrict the application of these products, in part due to the long-lasting legacy of copper-based algaecides and pesticides in aquatic systems [20], as well as the potential for adverse effects on non-target organisms [21,22]. For many other potential algaecides, development and testing can be hampered or even blocked because of permitting requirements under the US Federal Insecticide, Fungicide, and Rodenticide Act (FIFRA) and the National Pollutant Discharge Elimination System (NPDES), leading to exploration of algaecides that are based on natural products and therefore exempt from FIFRA requirements.

So-called natural algaecides have garnered considerable attention, given the potential for reduced impacts on non-target organisms, and can exhibit high efficiency and selectivity; they are often considered to be more ecosystem-friendly [23]. The majority of these compounds are derived from bacteria [23,24,25], but the literature is also rich regarding plant-based compounds such as essential oils [23,26,27,28,29], including for application to HAB organisms [30,31,32,33,34,35,36,37,38,39,40]. While many of these studies target a specific chemical, there has also been interest in compound products [30,41]. Despite this strong interest in green algaecides, a recent meta-analysis of various treatments used in freshwater systems identified only four effective chemicals, copper sulfate, hydrogen peroxide, peracetic acid, and simazine, while the bacterial, physical, or plant-based treatments did not significantly improve water quality [42]. Thus, despite testing numerous natural compounds, no single natural algaecide has emerged as a potential treatment that is as cost-effective, scalable, and efficient as traditional treatments, despite the desire to find an environmentally friendly alternative to traditional algaecides.

Anjon AG is a research and development company that specializes in organic pesticides for agriculture. Most of their agricultural products are registration-exempt under Sec. 25(b) and 40 CFR 152.25(f) of FIFRA. Based on this experience, the company has formulated an algaecide, C7X1, that uses a variety of FIFRA-exempt ingredients (Table 1) rather than a specific compound. This contribution provides a benchtop-scale assessment of C7X1 as a potential natural algaecide that meets the requirements (i.e., effectiveness, scalability, durability, ease of application, streamlined permitting processes) for water resource managers considering short-term mitigation of algal blooms [17].

This study assesses the efficacy of C7X1 at a laboratory scale using a variety of prokaryotic and eukaryotic algae, as well as with field-collected water from an inland lake that seasonally exhibits mixed assemblages of algae, often dominated by *Microcystis* [43]. Algal cultures were chosen primarily to represent HAB and non-HAB organisms endemic to California waters [44,45,46], including fresh, brackish, and marine organisms with representatives from the classes Bacillariophyceae, Coccolithophyceae, Cyanophyceae, and Raphidophyceae (see Methods). *Isochrysis galbana* was chosen as a representative non-HAB and *Gloeocapsa alpicola* as an organism with a distinctive mucosal sheath, which might confer some protection from algaecide exposure. The *Microcystis aeruginosa* strain used in this study was chosen based on consistent toxin production, ease of culturing, and use in other studies as a toxic *Microcystis* strain [47,48,49]. Harmful algae can exhibit considerable strain variability and plasticity under varying environmental conditions [50,51,52,53], so the results presented here should be considered relevant but not a definitive description of C7X1 efficacy against all algal classes.

## 2. Results

### 2.1. Exposure Concentrations for the Inhibition of Photosynthesis

The algal cultures and field samples were tested using a range of C7X1 concentrations to assess efficacy in suppressing photosynthesis. The concentration of algaecide resulting in 50% inhibition of maximal F_v_/F_m_ (IC_50_ values) and the experimental treatment where F_v_F_m_ reached a value of <0.05 (complete shutdown of photosynthesis) were comparable across algae in benchtop testing, with IC_50_ values of approximately 400–14,000 ppm C7X1 and complete shutdown of photosynthesis within 24 h ranging from 4000 to 20,000 ppm (Table 2). Testing on water obtained from Pinto Lake, CA, provided comparable results. At time 0 (immediately after adding C7X1), F_v_/F_m_ dropped to <0.05 at concentrations of 6250 ppm and higher for Pinto Lake water, demonstrating that C7X1 is capable of nearly instantaneous suppression of photosynthesis at sufficiently high concentrations.

### 2.2. Reduction in Chlorophyll and Cell Counts

**Figure 1:**
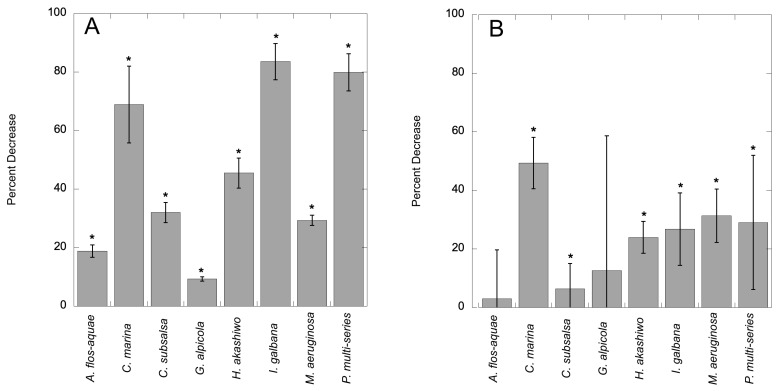
Percent decrease in chlorophyll after exposure to C7X1 at 7000 ppm for 24 h (**A**) and percent decrease in cell counts after exposure (**B**). Error bars are standard deviations from six biological replicates for Panel A and three biological replicates for Panel B. Statistically significant decreases (Student’s *t*-test, *p* < 0.05) are indicated with asterisks (*).

**Figure 2:**
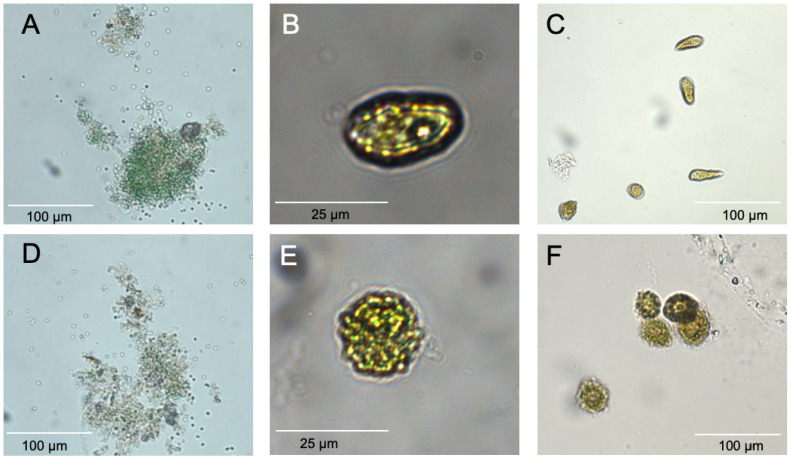
Micrographs of representative algae exposed to 7000 ppm C7X1 for 24 h. (**A**) *M. aeruginosa* control; (**B**) *H. akashiwo* control; (**C**) *C. marina* control. (**D**) *M. aeruginosa* treated with algaecide; (**E**) *H. akashiwo* treated with algaecide; (**F**) *C. marina* treated with algaecide. Each pair of micrographs was imaged with the same magnification and illumination.

Cultures exposed to 7000 ppm C7X1 for 24 h exhibited a decline in chlorophyll ranging from 9.5 to 85% (Figure 1A) and noticeable deformation and loss of pigmentation in some of the cultures (Figure 2). The decrease in chlorophyll for each algal group tested was significant at *p* < 0.05 (Student’s *t*-test). All cultures exhibited decreases in mean cell counts as well (Figure 1B) but with a lower percent range of 3–49% and with six of eight cultures demonstrating significant reductions (*p* < 0.05) in cell counts; *A. flos-aquae* and *G. alpicola* were not significant with *p*-values of 0.16 and 0.26, respectively.

### 2.3. Effect of Algal Biomass on C71X Efficacy

**Figure 3:**
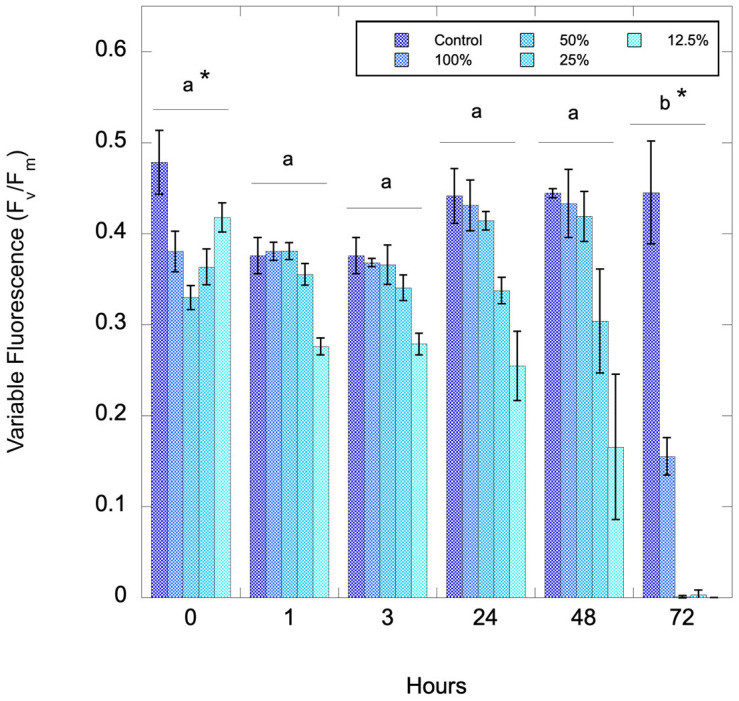
Variable fluorescence (F_v_/F_m_) from Pinto Lake that was serially diluted and exposed to 1000 ppm C7X1, with sampling conducted from 0 to 72 h. Error bars are the standard deviation of three biological replicates. There were significant differences for the 72-h timepoint (b) versus all other timepoints (a) and between the Control and 12.5% dilution (*).

Pinto Lake water from 28 August 2024 was serially diluted to assess whether there is a biomass effect with the algaecide and to assess the effect of time of exposure to the algaecide. Figure 3 shows a timeseries after treatment with 1000 ppm C7X1. There were significant differences with both dilution (amount of biomass, *p* = 0.005) and time (*p* < 0.001, ANOVA with Tukey Honestly Significant Difference post-hoc test).

**Figure 4:**
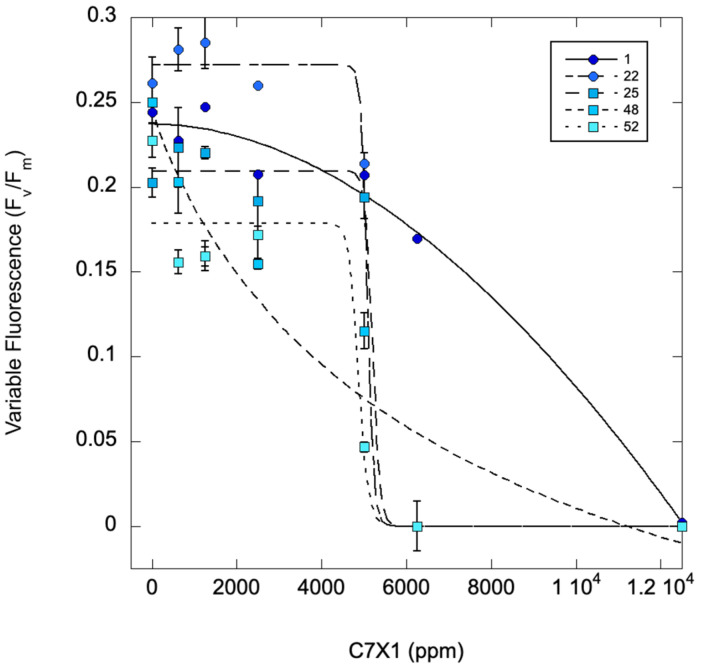
Variable fluorescence (F_v_/F_m_) for *M. aeruginosa* exposed to varying concentrations of C7X1 and assessed from 1 to 52 h after treatment with algaecide. Error bars represent standard deviations from three biological replicates. IC50 values were 7341, 5208, 5092, 4885, and 3466 ppm from 0 to 52 h.

Figure 4 shows an *M. aeruginosa* culture treated with C7X1 at 0 (control), 625, 1250, 2500, 5000, 6250, and 12,500 ppm and measured sequentially at 0, 1, 22, 25, 48, and 72 h. The IC_50_ values steadily decrease with time, ranging from 7341 ppm (0 h) to 3466 ppm (48 h) ppm C7X1. There also appeared to be a threshold response with a rapid decrease in F_v_/F_m_ above 2500 ppm. Based on these results, subsequent tests typically used 24-h exposure for IC_50_ values (Table 2) for intercomparison of the results.

### 2.4. Vertical Column Experiments

A typical deployment scenario for the use of algaecide is to spray it onto the surface of a water column. To assess how C7X1 would perform in that scenario (at lab scale), 0.5 m, 1 L vertical Plexiglas columns were filled with either Pinto Lake water collected on 18 August 2024 and 28 September 2024 or *H. akashiwo* culture, which was chosen as it is a strong vertical migrator.

#### 2.4.1. Pinto Lake, 18 August 2024

For the first experiment, the water was mixed, dispensed into four columns, and allowed to equilibrate for 24 h in an environmental chamber. The chlorophyll concentration at the time of sampling was 45.4 µg L^−1^. Initial F_v_/F_m_ was 0.348 (±0.03), consistent with a moderately healthy community dominated by cyanobacteria. The IC_50_ for the whole water was 3179 ppm (SE = 370). Zooplankton activity (movement, feeding) did not appear to be impacted by C7X1 below concentrations of about 4000 ppm.

**Figure 5:**
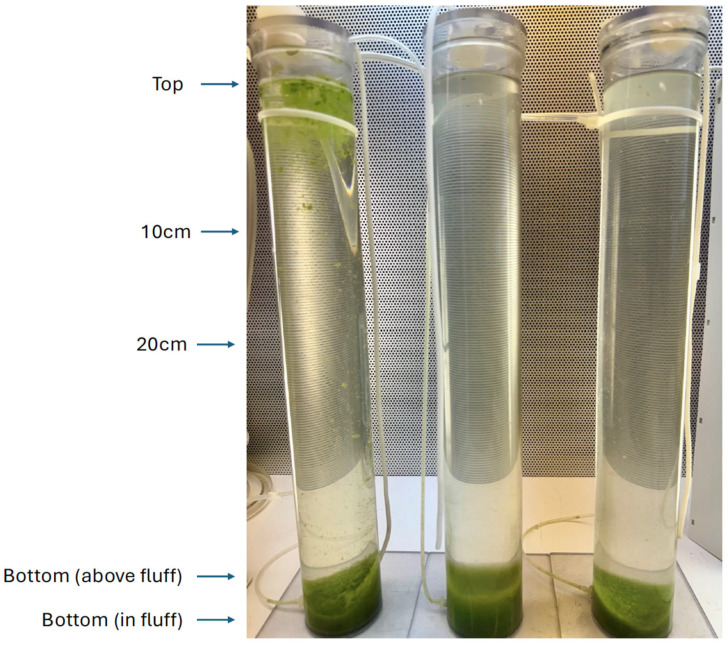
Vertical column experiments with Pinto Lake water at 48 h after treatment. Left: Control; Middle: treated and mixed; Right: treated at the surface only, the C7X1 is visible as an off-white layer at the top of the column. Annotations on the left indicate depths at which samples were collected. The fourth column (mixed and treated) was omitted from the photo but was comparable to the treated columns depicted in the figure.

At the start of the experiment, each column was separated into a surface layer, diffuse cells throughout the column, and a bottom layer of cells (Figure 5). The Control column sampled at the top had an rETR_max_ value of 325.2 ± 38.4. One column was not treated (Control), and the other three were treated at a concentration of the equivalent of 5000 ppm for the full column volume. Column 2 was mixed before spraying the C7X1 at the top of the column, Column 3 was treated and then mixed, dispersing the C7X1 through the full volume, and Column 4 was sprayed at the surface with no mixing. While the density difference between C7X1 and either fresh or saltwater is enough to form a layer (see the treated but not mixed column in Figure 5), it is miscible in both and was observed to gradually mix/diffuse downward.

The columns were sampled after 1.5 h and resampled after 24 and 48 h at the top, 10 cm, 20 cm, immediately above the biomass at the bottom of the column and from within the bottom layer (Figure 5). Samples were analyzed for F_v_/F_m_ and rETR curves. At the termination of the experiment, the Control column was treated with 5000 ppm C7X1 and mixed, replicating Column 3.

At 1.5 h, the Control F_v_/F_m_ was 0.357 (±0.02) or healthy for a cyanobacterial bloom. The treated columns exhibited depressed F_v_/F_m_ at the surface, F_v_/F_m_ < 0.1, and ETR curves were not plotted since there was essentially no fluorescence signal. Column 3 exhibited no fluorescence (F_v_/F_m_ < 0.05), and no additional sampling was conducted. The Control column (replicating Column 3 at the end of the experiment) also showed no variable fluorescence after treating and mixing the column.

**Figure 6:**
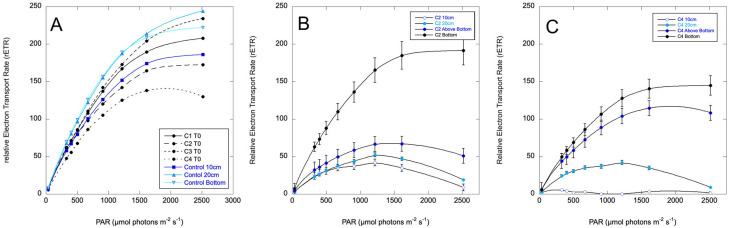
Relative Electron Transport Rate (rETR) curves for the columns prior to treatment (**A**), Column 2 (C2), which was mixed and then treated by spraying C71X at the top (**B**), and Column 4 (C4), which was sprayed at the top with no mixing (**C**). Depth sampling for C2 and C3 is as indicated in Figure 4. Error bars are standard deviations for three biological replicates.

Variable fluorescence was negligible on the surface of the treated columns at 24 h. Photosynthetic performance is plotted as rETR curves in Figure 6 for sampling at 48 h. The maximum rETR was reduced by about half throughout the column, with progressively more impairment approaching the surface. The mixed and treated column (Column 2) was more uniform, with the least impairment in the bottom layer of flocculent material. Column 3, which was not mixed, exhibited a stronger vertical gradient of impairment. This is potentially due to the C7X1 being more evenly distributed in Column 2 compared to Column 3, as mixed cells sank out in Column 2. The C7X1 was presumably more gradually mixed downward in Column 3 as buoyant cells sank from the surface, where the algaecide had been sprayed. At 72 h, all the treated columns were clear of cells except for the bottom flocculant material. The columns remained clear through day 7, when the experiment was terminated.

#### 2.4.2. Pinto Lake, 28 September 2024

The second column experiment was set up in an identical fashion, except that the treated and mixed column was omitted. Initial F_v_/F_m_ was 0.457 (±0.01). Chlorophyll concentration at the time of sampling was 80.2 µg L^−1^. The IC_50_ was 8832 (SE = 2932). Columns were again treated with 5000 ppm C7X1. However, except for the Control, all columns immediately cleared of cells, and variable fluorescence dropped to <0.05 through the full vertical extent. The experiment was therefore terminated without additional rETR curves, and the columns were maintained to determine whether there would be recovery. None but the control exhibited positive F_v_/F_m_ values at 7 days.

When the unmixed column was sprayed with C7X1, the upper 10 cm immediately developed a blue tint consistent with cell lysis and release of phycocyanin. Given the rapidity of the response and the much lower maximum rETR in September of 78.1 ± 8.0 compared to August (325.2 ± 38.4), it is likely that the bloom in Pinto Lake was senescing and therefore more susceptible to the algaecide compared to the August experiment.

#### 2.4.3. *Heterosigma akashiwo* Cultures

A third set of column experiments was conducted with *H. akashiwo* strain EBL 71. Columns were Control (replicates) and treated (replicates) with surface spray and no mixing. Based on IC_50_ values for *H. akashiwo*, treatment was reduced to 1000 ppm C7X1. Initial F_v_/F_m_ of the stock culture indicated that the cells were healthy (F_v_/F_m_ = 0.54 ± 0.02) but below optimal values of ~0.65. At the start of the experiment, cells had migrated to the upper third of the columns. Photosynthetic responses, as rETR curves, were measured at the beginning of the experiment (before treatment) and at 24 h.

**Figure 7:**
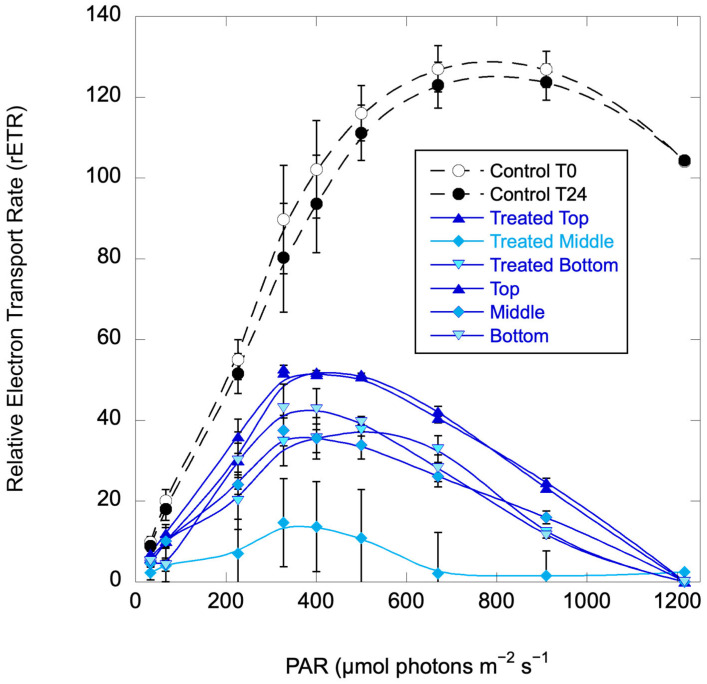
Relative Electron Transport Rate (rETR) curves for the *H. akashiwo* control (untreated) columns at the initiation of the experiment (T0) and at 24 h, and for replicate treated columns at 24 h. Error bars are standard deviations for replicate columns (control) or three replicates from each of two treated columns.

At 24 h, the treated columns exhibited greatly reduced photosynthesis compared to the control columns (Figure 7) with uniform reductions (except for one treated column sampled at 20 cm, or the middle of the column, which was even more suppressed). At 48 h, the control columns were still healthy but the treated columns were cleared, with all cells on the bottom and no photosynthetic activity.

#### 2.4.4. Pinto Lake Large Volume Columns

The last set of experiments was scaled up to 1.5 m, 20 L volume Plexiglas columns that were maintained in an environmental chamber. The initial chlorophyll was 5.49 µg L^−1^, and the experiment was designed to mimic pre-bloom conditions. BG11 media at 0.1% of stock concentration (17.6 µM N, 0.23 µM P) was added to all columns. Replicate treatment columns were sprayed with the equivalent of 1000 ppm for the 20 L volume at the top of the column. The initial F_v_/F_m_ was 0.432 (±0.033) or healthy for a mixed assemblage with cyanobacteria. At one week, the control column F_v_/F_m_ was still healthy at 0.447 (±0.018), while the treated columns were reduced to 0.136 (±0.002) at a 20 cm depth. At two weeks, F_v_/F_m_ increased in the controls to 0.509 (±0.017) while the treated columns increased to 0.247 (±0.02). At one month, the experiment was terminated, with F_v_/F_m_ of 0.498 (±0.065) and 0.225 (±0.004) for the control and treated columns. The control columns had a pronounced surface accumulation of algae, dominated by *Volvox* colonies. The treated columns had a surface layer of senescent and unhealthy *Volvox*, and the water column was dominated by detritus. Final chlorophyll concentrations in the control and treated columns, sampled at 10 cm to avoid the surface mats, were 2.25 (±1.48) and 27.5 (±26.1) µg L^−1^ chlorophyll.

### 2.5. Toxin Release

**Figure 8:**
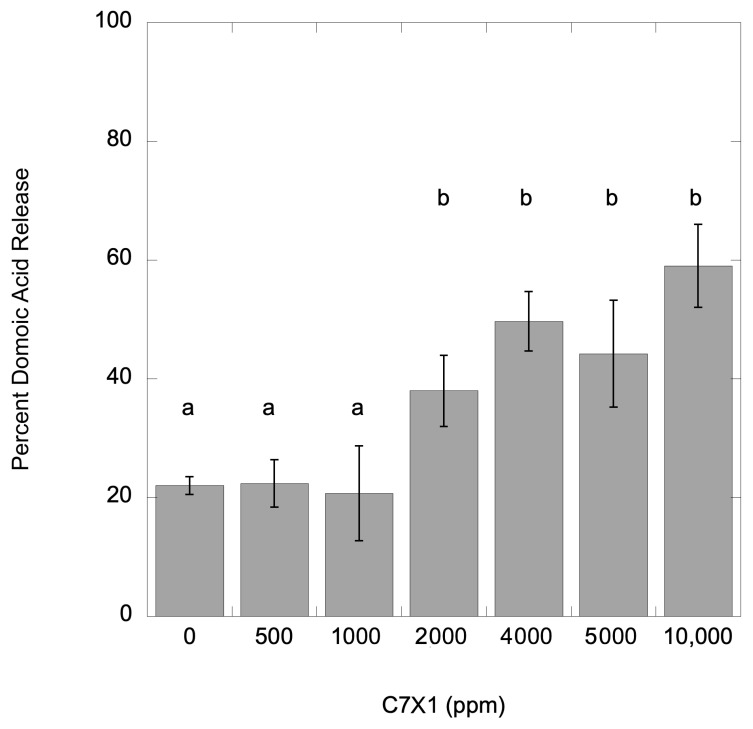
Percent toxin release from *P. multiseries*. Error bars are the standard deviation from three replicates; letters indicate significantly different groupings (Tukey HSD, *p* < 0.05).

For the *M. aeruginosa* and *P. multiseries* cultures, samples from the IC_50_ experiments were sampled for total and dissolved (released) toxin concentrations for microcystins and domoic acid, respectively. Data for the toxin release experiments are presented as a percent dissolved toxin. The control (no C7X1 treatment) samples had 22.0% (±1.5%) dissolved toxin and 885.0 (±55.2) µg L^−1^ total toxin. Replicates were analyzed after exposure for 24 h to 500, 1000, 2000, 4000, 5000, and 10,000 ppm C7X1. Results are presented in Figure 8. There was no significant difference from the control up to 1000 ppm C7X1 (*p* < 0.001) and 58.9% (±6.9%) release at the highest dose of 10,000 ppm. Doses greater than 1000 ppm resulted in significantly more domoic acid release (*p* > 0.05).

**Figure 9:**
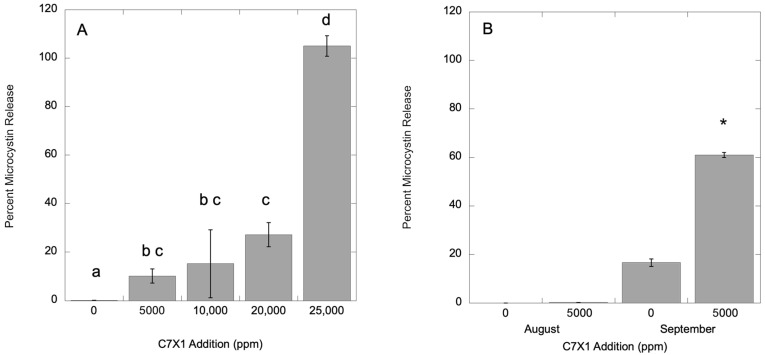
Percent toxin release from *M. aeruginosa* culture (**A**) and Pinto Lake water (**B**) from column experiments in August (left) and September (right). Error bars are the standard deviation from three replicates, letters (panel (**A**)) and asterisks (panel (**B**)) indicate significantly different groupings (Tukey HSD, *p* < 0.05).

For *M. aeruginosa*, there was negligible dissolved microcystin in the control (0.1%) with 33.2 (±1.59) µg L^−1^ total microcystin. Replicates were analyzed after exposure for 24 h to 5000, 10,000, 20,000, and 25,000 ppm C7X1. Toxin release increased with increasing algaecide additions, with significant increases at 5000, 20,000, and 25,000 ppm (*p* < 0.05). The results are presented in Figure 9A. Samples were also analyzed from the column experiments on 18 August 2024 and 28 September 2024 using Pinto Lake water, presented in Figure 9B. The August control had 635.9 (±49.6) µg L^−1^ total microcystins with 0% in the dissolved phase. The September control had 23.7 (±0.90) µg L^−1^ total microcystins with 16.6% in the dissolved phase. After 24 h exposure to 5000 ppm C7X1, dissolved toxins did not change in the August experiment, but increased significantly to 60.9% in the September experiment, and were at the same concentration at the top and bottom of the columns. As noted above, there appeared to be release of phycocyanin in the treated columns from September, consistent with cell lysis after treatment with C7X1 despite the relatively high F_v_/F_m_ value for the control of 0.457 (±0.01) and lack of lysis in either the *Microcystis* culture or the Pinto Lake water from August.

### 2.6. Stability of the Algaecide

**Figure 10:**
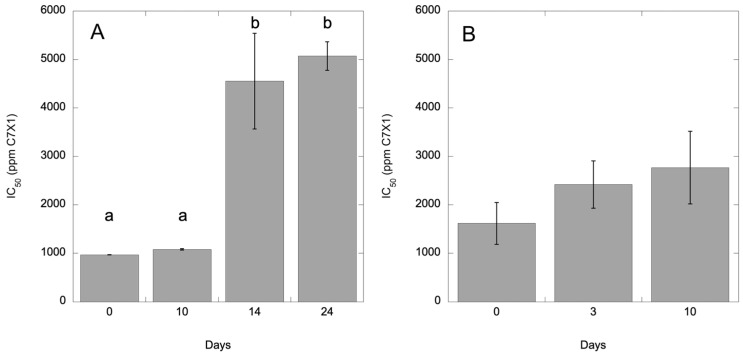
IC_50_ for (**A**) *I. galbana* and (**B**) *H. akashiwo* treated with C7X1 that was exposed to natural sunlight in UV-transparent quartz tubes for 0–24 days (**A**) and 0–10 days (**B**). Error bars are the standard deviation of three biological replicates. Letters indicate significantly different groupings (Tukey HSD, *p* < 0.05).

As per manufacturer recommendations, C7X1 was stored at room temperature. Repeated experiments at 0-, 3-, and 6-months using *I. galbana* culture were 5314 (563), 969 (1), and 4085 (1150) for IC_50_ (SE). While the IC_50_ values varied significantly (ANOVA, *p* < 0.05) at the second time point, there was no consistent increase in IC_50_ values with time compared to shorter term experiments where UV degradation was expected (Figure 10), suggesting that the observed variability was related to the physiological state of the culture at each time point rather than storage of C7X1. To assess whether it would degrade under simulated environmental conditions, C7X1 was diluted 1:10 with filtered seawater and placed in a UV-transparent quartz glass tube. The tube was left outside to expose it to fluctuating temperatures and UV light. The C7X1 was used for IC_50_ curves using *I. galbana*. Figure 10 shows the gradual degradation of efficacy, resulting in increasingly higher IC_50_ values with a significant difference at 14 days (ANOVA, *p* < 0.05). A similar but truncated experiment was performed using C7X1 diluted 1:10 with 0.2 µm filtered Pinto Lake water. *H. akashiwo* was treated with the control and UV-treated (Pinto Lake water) algaecide at 0, 3, and 10 days. At 3 days, there was no difference between UV and non-UV C7X1. At 10 days, the IC_50_ increased from 1618 ppm to 2769 ppm, or about double, compared to *I. galbana*, which increased about 10% at the same timepoint but was not significant compared to the control treatment (ANOVA, *p* = 0.053).

## 3. Discussion

The purpose of this study was to assess the efficacy of a pre-commercial algaecide based on FIFRA-exempt natural products. The algaecide, C7X1, produced by Anjon AG, was assessed against a broad but not exhaustive group of algal cultures, focusing primarily on HAB organisms found in California waters. Laboratory culture experiments were supplemented with field samples collected from a nearby lake with a history of cyanobacterial HAB issues [54]. The primary metric for assessment of efficacy was reduction in variable fluorescence, generally considered to be a good indicator of overall cell health. The C7X1 formulation was effective against all algae tested, but with varying IC_50_ levels, suggesting that individual species and possibly strains have varying responses to the algaecide. For *Microcystis* and *Pseudo-nitzschia*, the extracellular release of toxin increased with the increasing dose of algaecide but resulted in less than 100% release when dosed at or below the IC_50_ level.

### 3.1. Exposure Concentrations for Inhibition of Photosynthesis

The C7X1 algaecide exhibited a range of IC_50_ values and lethal doses depending on the algal strain (Table 2). An effective algaecide must be concentrated enough that application to large waterbodies is reasonable. For comparison, Kinley-Baird et al. [17] reported minimum effective exposure concentrations for a broad range of commercially available algaecides as mg L^−1^ of active ingredient. C7X1 is 20.1% active ingredients (Table 1) with a density of 925 mg L^−1^, so 1000 ppm (*v*/*v*) would be approximately equivalent to 0.19 mg L^−1^ active ingredients.

Kinley-Baird et al. [17] assessed the dose based on the decline in cell densities and chlorophyll a rather than Fv/F_m_ but the values reported ranged from 0.51 to 1.01 mg L^−1^ for the copper-based algaecides, 11.4 mg L^−1^ for the peroxide-based algaecides, and 0.139 mg L^−1^ for endothall acid. Zhou et al. [32] tested Diuron and ethyl 2-methylacetoacetate (EMA) on *Microcystis* and identified significant declines in cell biomass at 48 and 72 h with exposure to 0.75 mg L^−1^ Diuron and 500 mg L^−1^ EMA, respectively. C7X1 IC_50_ values ranged from 0.076 to 2.57 mg L^−1^, so within the range of effective dose for commercially available treatments.

An advantage of C7X1 compared to many other compounds is that it is FIFRA-exempt and will not contribute to legacy contamination compared to copper-based treatments. For that reason, there has been considerable interest in identifying plant-based algaecidal compounds. Zhao et al. [33] identified the plant-based alkaloids nefirine and nuciferine at concentrations of 0.25–4.5 mg L^−1^ to be effective against *Microcystis*, with a half-maximal effective concentration (EC_50_) of 0.52 mg L^−1^. Other natural products have also been considered, including mixtures of compounds from Chinese traditional medicines [37]. A combination of compounds from golden thread and areca seed was most effective, at a dose of 0.048% (*w*/*v*) or 480 mg L^−1^. For C7X1, the comparable concentration to achieve a similar reduction, noting that each study used different metrics for impairment, would be 0.19 mg L^−1^. C7X1 thus provides comparable results to other plant-based compounds, as indicated by reduced photosynthetic performance at similar concentrations with significant reductions in chlorophyll at 24 h (Figure 1).

### 3.2. Mode of Action for Inhibition

Pulsed Amplitude Fluorometry has been proposed as a convenient and sensitive metric for evaluating algaecide performance [32] and has been widely used to assess a broad range of compounds as algaecides [55,56,57,58]. Variable fluorescence is generally considered to be an indicator of overall photosynthetic performance, with lower values representing a response to environmental stressors. In general, a decrease in F_v_/F_m_ represents reduced photosynthetic capacity. All the algae showed a decrease in F_v_/F_m_ with exposure to C7X1, consistent with a negative response to the active ingredients that directly impacts photosynthetic performance. Examining Figure 6 and Figure 7, exposure to C7X1 decreased the initial slope (α) as well as resulting in an increase in photo-inhibition (β). At the same time, rETR_max_ decreased compared to the controls. A reduction in rETR_max_ can result from lowering electron transport chain and Calvin cycle activities [59], suggesting that the algaecide impaired photosystem II (PSII). The simultaneous decline in α and rETR_max_ and increase in β are all consistent with general impairment of the photosystem, likely at multiple points in the photosynthetic process.

Visual examination of cells by microscopy (Figure 2) showed the distortion of shape and bleaching or a reduction in pigments, but cells remained intact. A reduction in chlorophyll over 24 h suggests that pigments were being destroyed or catabolized within the cell, which is consistent with the changes in α and β. The reduction in chlorophyll was consistently greater than the reduction in cell density, supporting the proposed physiological responses. All of these results are consistent with C7X1 damaging the photosynthetic apparatus and likely resulting in cellular oxidative stress [60], similar to other plant-derived natural products tested as algaecides [33].

### 3.3. Extent of Toxin Release After Exposure to C7X1

Minimizing algal toxin release is a potential priority in management actions where blooms are near drinking water intake sites or there is potential for the use of contaminated water in agricultural applications [61,62,63,64]. Much less is known about the potential threat of dissolved domoic acid in marine and estuarine environments, but studies show that it can be ubiquitous in estuarine and coastal marine environments and is likely related to low-level contamination of bivalves [11,65].

While human and environmental health risks are more clearly associated with total toxin load rather than dissolved toxin [17], the ability to clear the water column, as shown in the vertical column experiments, and sequester biomass with minimal release of toxins is preferential for an effective algaecide [66]. Benchtop testing with *M. aeruginosa* and *P. multiseries* cultures demonstrates that there is low to moderate toxin release at concentrations of C7X1 at the IC_50_ treatment level and that very high concentrations are required to approach full release for healthy cells (Figure 8 and Figure 9).

Column experiments in August and September resulted in flocculation of the cells and sequestration of toxin to the bottom of the column in the first set of experiments (August) but considerable release with uniform distribution of dissolved microcystins in the second set of experiments (September). While this is likely related to the overall health of the assemblages in August and September, more testing at mesocosm or contained field trial scales is warranted to determine whether C7X1 can both clear the water column and sequester cells and toxins at depth to develop management strategies that both visibly mitigate algal blooms and protect the public from inadvertent exposure to dissolved toxins [66].

### 3.4. From Bench Scale to Environmental Testing

Laboratory results using cultures and Pinto Lake water at up to 20 L volumes suggest that C7X1 is a promising algaecide that is comparable to both commercially available and compound-specific formulations. If C7X1 were to move from bench-scale testing to environmental application, these results provide some guidelines for use and further testing. The column experiments demonstrate that C7X1 will penetrate vertically without mixing, making the algaecide amenable to surface spray application. The efficacy of C7X1 is both biomass-dependent (Figure 3) and organism-dependent (Table 2), such that some basic knowledge of the system where it is applied is required to optimize application. The 20 L column experiment, while not exposed to UV, did demonstrate the ability to suppress bloom conditions after one month, with an approximately 10-fold reduction in surface biomass relative to the control and no change in community composition but with an increase in detritus and senescent algae. C7X1 has a reasonably long decay time when tested with UV exposure and is shelf-stable when not exposed to UV, suggesting that repeat application would be required on timescales of weeks. While off-target effects were not specifically addressed in this study, qualitative examination of the zooplankton community from the Pinto Lake experiments suggest that at doses near the IC_50_ range, zooplankton activity and abundance were not reduced but it should be noted that zooplankton can exhibit substantial intraspecific variability in response to toxicological stressors [67] and the qualitative results reported here are not necessarily representative of impacts to other organisms [68].

### 3.5. Future Directions

The evaluation of C71X indicates potential as a natural plant-based algaecide that could be commercialized. While this study tested several algal genera, further assessment of the efficacy of C7X1 with other algal groups would be valuable. No dinoflagellate cultures were included in this study, although *Ceratium* was present in the field-collected water. Testing of representative high-biomass dinoflagellates such as *Karenia brevis* [69] and *Akashiwo sanguinea* [70] would be relevant.

For the cultures that were tested in this study, the primary metric for assessing efficacy was suppression of photosynthesis over short timescales (24 h to several days). The results demonstrated that there was no recovery in either the column experiments or the benchtop testing, leading to the conclusion that F_v_/F_m_ values <0.05 are indicative of cell death, but many other bench-scale studies of potential algaecidal compounds include reduction in cell density or changes in growth rates as metrics [17]. For this study, short-term (24 h) reduction in cell density was assessed. Additional experiments testing whether there are long-term changes in growth rates, reductions in cell density, and/or development of resistance to sublethal doses of C7X1 would be beneficial. Additional testing could also incorporate vital staining to assess cellular permeability and cell mortality [57].

More information on extracellular toxin release after exposure to C7X1, particularly using natural assemblages rather than cultures, would also be of benefit. The results from this study show low to moderate release of toxins in culture (Figure 8 and Figure 9) but with variable results when testing field samples (Figure 9). Repeated sampling of a field assemblage as a bloom senesces, in combination with vital staining, would clarify whether senescing cells are more likely to release intercellular toxins.

This study provides laboratory-scale analysis of C7X1 performance as a potential algaecide, including appropriate dosage for multiple algal groups and evidence that surface application can be effective for a water column. Use of C7X1 at scale in the field will be required to answer remaining questions about the frequency of application and real-world outcomes.

## 4. Conclusions

The overall objective of this study was to provide preliminary data on the efficacy of the algaecide C7X1 with bench-scale testing, prior to demonstration-scale and full application of the algaecide. The C7X1 formulation is effective against a wide range of algae, results in minimal toxin release, and at bench scale, is appropriate at high concentrations for the immediate suppression of blooms or at lower concentrations for longer-term suppression of algae.

The frequency and intensity of harmful algal blooms are increasing both within the United States and globally, increasingly requiring management strategies that include active mitigation [4]. C7X1 provides a “green” alternative to other USEPA-registered algaecides and could potentially be used in waterbodies, streamlining permitting, since all ingredients are exempt under FIFRA. While many other algaecide studies have focused on freshwater HABs such as Microcystis, the applicability of C7X1 to freshwater, estuarine, and marine HABs highlights the potential for broad application if logistical and management challenges that emerge at scale can be overcome.

## 5. Materials and Methods

### 5.1. Algaecide Source, Storage, and Degradation

The C7X1 algaecide was provided by Anjon AG (Weatherford, TX, USA). The algaecide was stored per the manufacturer’s directions at room temperature in polyethylene containers and vigorously shaken before dispensing. It was generally kept in the dark, but the manufacturer’s instructions do not require dark storage. To test whether the algaecide degraded with time, IC_50_ tests (see Section 5.5) were repeated through time at 0, 3, and 6 months using *Isochrysis galbana*, with IC_50_ values compared as a function of time. To assess potential degradation under simulated environmental conditions, C7X1 was diluted 1:10 with sterile filtered seawater and placed in a UV-transparent quartz glass tube. The tube was left outside for 24 days, and the C7X1 was used for IC_50_ curves using *Isochrysis galbana* conducted at 0, 10, 14, and 24 days. At each treatment time, laboratory-stored C7X1 was used as a control with the same cultures. A second experiment was conducted with 0.2 µm filtered Pinto Lake water using a Cytiva Whatman GD/X syringe filter (Marlborough, MA, USA), part number 6870-2502, to remove bacteria but leave other chemical compounds such as tannins, etc., intact. The C7X1 mixed with Pinto Lake water and exposed to UV was tested with *Heterosigma akashiwo* at 0, 3, and 10 days. The algaecide was miscible in both fresh and saltwater. C7X1 density was determined gravimetrically using an OHAUS Explorer analytical balance (OHAUS, Parsippany, NJ, USA).

### 5.2. Algal Strains

Testing of C7X1 was performed using both monospecific algal cultures (not axenic; Table 2) and with field samples. Cultures used in this study included *Microcystis aeruginosa*, *Gloeocapsa alpicola*, *Aphanizomenon flos-aquae*, *Heterosigma akashiwo*, *Pseudo-nitzschia multiseries*, *Isochrysis galbana*, and *Chattonella* spp. (Table 3). Freshwater strains were maintained at room temperature (~20 °C) and in ambient light on either Bold 3N or BG-11 media obtained from the University of Texas Culture Collection (Austin, TX, USA). Estuarine species were maintained on Guillard’s f/2 or L1 media obtained from the Provasoli-Guillard Culture Collection (Bigelow, ME, USA); cultures were maintained at 32 PSU for all but *H. akashiwo*, which was at 24 PSU and at 15 °C or 20 °C (Table 3) with 150 µmol photons m^−2^ s^−1^ light and 12:12 light:dark in environmental chambers. All experiments were conducted with algae in the exponential or early stationary growth phase, and nutrients were replete throughout the experiments. A subset of cultures were documented with and without exposure to C7X1 at doses sufficient to inhibit photosynthesis using a Zeiss Axio Imager A.1 microscope (Oberkochen, Germany) equipped with a Teledyne Lumenera (Richmond, BC, Canada) color camera. Typical growth rates, obtained from daily fluorescence measurements for at least three replicate cultures, are reported in Table 2.

### 5.3. Effects of C7X1 on Chlorophyll and Cell Density

Cultures were exposed to 7000 ppm C7X1 for 24 h in two separate experiments. At 24 h, samples were collected for chlorophyll content from control and treated samples (three replicates each) and analyzed following a non-acidification extraction method [71] using a Turner Designs 10AU fluorometer (San Jose, CA, USA) calibrated with pure chlorophyll *a* from Sigma-Aldrich, St. Louis, MO, USA, part # 96145). For the second experiment, cells were enumerated by microscopy using either a nanoplankton counting chamber or a hemocytometer and the Zeiss Axio Imager A1 described above, with three replicates for control and treated cells. Growth rates were calculated using Equation (1), as follows:

µ = t^−1^ × ln(N_t_/N_0_) (1)

where *µ* = growth rate (d^−1^), *t* = time, *N*_0_ = biomass at the beginning of exponential growth, and *N_t_* = biomass at the end of incubation, with the interval being a minimum of three days. All fits had r^2^ values > 0.9.

### 5.4. Field Water Collection

Water was collected from Pinto Lake, CA. Pinto Lake is a shallow natural lake located 8.3 km inland from Monterey Bay and is well-characterized through an ongoing ~weekly time-series occupied from 2009 to the present [11,54]. Whole water was collected on 18 August 2024, 28 September 2024, and 28 January 2025. At the time of collection, nitrate concentrations were 1.7, 9.0, and 6.6 µM, while orthophosphate concentrations were 5.8, 10.2, and 14.1 µM (determined using flow-injection analysis with an autoanalyzer as described in [54]). See Appendix A for additional environmental data.

For each collection, surface (<1 m) water was collected in a 20 L polycarbonate carboy and transported to the lab, where it was maintained at ~100 µmol photons m^−2^ s^−1^ light (12:12 light:dark) and 15 °C in an environmental chamber. Samples were inspected visually using a Leica MZ 12.5 dissecting equipped with dark field scope (Deerfield, IL, USA), and community composition was quantified using a relative abundance scale: Rare (R): <1%; Present (P): 1–<10%; Common (C): 10–<25%; Abundant (A): 25–<50%. The assemblages were *Dolichospermum* (A) and *Ceratium* (A) for July, *Microcystis* (A) with low levels (P) of *Dolichospermum* and *Aphanizomenon* in August, *Microcystis* (A) with moderate levels (C) of *Dolichospermum* and low levels (P) of *Aphanizomenon* and *Ceratium* in September, and *Aphanizomenon*, *Microcystis* (C), and *Ceratium* (R) with bacteria and detritus making up the rest of the assemblage. Chlorophyll levels were 16.3, 59.6, 78.8, and 4.5 µg L^−1^, respectively. Zooplankton were not enumerated, but presence and activity were noted. Zooplankton included copepods, *Bosmina*, *Daphnia*, and rotifers.

### 5.5. Experimental Treatments for IC_50_

Algal biomass was assessed using relative fluorescence units (RFUs) on a Turner Designs 10AU fluorometer (Turner Designs Inc., San Jose, CA, USA), with RFU converted to equivalent chlorophyll using calibrations between RFU and acetone-extracted chlorophyll measurements [71]. Algal samples were diluted to approximately 50 µg L^−1^ chlorophyll with 0.2 µm filtered base water (using media for cultures and 0.2 µm filtered Pinto Lake water for field experiments; a Cytiva Whatman GD/X syringe filter (Marlborough, MA, USA), part number 6870-2502, was used for filtration) except for one test with Pinto Lake water collected 18 August 2024 where the water was serially diluted from 59.6 µg L^−1^ to 29.8, 14.9, and 7.5 µg L^−1^ to determine if there was a biomass effect with exposure to algaecide. For exposure to algaecide, 5 mL of water was subsampled into 10 mL borosilicate glass tubes and C7X1 was added at varying concentrations, with all concentrations reported as ppm or µL L^−1^ (*v*/*v*). The primary metric for assessment was change in variable fluorescence (F_v_/F_m_) after 24-h exposure, but a subset of experiments were sampled at both shorter (hours) and longer (days) timepoints to assess the temporal impact of the algaecide. Treatments were conducted in replicate or triplicate, and control samples (no algaecide) were included for each experiment. Replicates rather than triplicates were used when samples were lost, when there was inadequate equipment or incubator space to conduct triplicate experiments (vertical columns), or when serial replicates requiring a single piece of equipment (e.g., WATER-PAM or vertical columns) would result in unacceptably long experimental measurement time.

Algaecide was added with or without 10-fold dilution using base water so that no more than 5% of the 5 mL test volume was added for each treatment. Control samples had the same volume of deionized water added. Treated samples were maintained under the same conditions as the Pinto Lake water or cultures for up to 7 days.

### 5.6. Inhibitory Concentration Curves and Statistics

The F_v_/F_m_ data were used to calculate the half-maximal inhibitory concentration (IC_50_) at 24 h and the dose at which photosynthesis was completely inhibited (F_v_/F_m_ < 0.05). IC_50_ values were determined in Kaleidagraph v. 5.01 (Synergy Software, Reading, PA, USA) using a modified dose–response curve:

y = a + b/(1 + (x/c)^d^) (2)

where x = concentration of C7X1, y = the F_v_/F_m_ value, a = the minimum response value, b = the range of transition (y_max_ − y_min_), c = IC_50_, and d = the slope of the fit. Error estimates are the standard error of the model fit for each variable. All fits had r^2^ values > 0.9.

Eukaryotic algae generally exhibit a y_max_ value of ~0.65 (healthy) to 0 (no photosynthetic response). Cyanobacteria typically exhibit lower y_max_ values (~0.3–0.4) for healthy cells when using 660 nm red excitation light on the WATER-PAM [72]. Repeated IC_50_ values at multiple time points were used to assess the degradation of C7X1 (loss of efficacy) after exposure to ambient outdoor environmental conditions in a sealed quartz tube for up to 24 days, allowing penetration of UV light into the stock solution and natural oscillations in irradiance and temperature. Experiments were conducted in January 2025 in Santa Cruz, CA, USA.

A comparison of differences between groups was conducted in Kaleidagraph v. 5.01 (Synergy Software). For comparison of two factors, two-tailed Student’s *t*-test distributions were used. For comparison using multiple factors, one-way and two-way ANOVA were used, with a Tukey Honestly Significant Difference (HSD) post hoc test for ANOVA results that were statistically significant. Significance was set at a value of 0.05.

### 5.7. Assessment of Variable Fluorescence and Electron Transport Rates

The primary metric for assessing the efficacy of C7X1 was through measurement of variable fluorescence (F_v_/F_m_) and relative Electron Transport Rates (rETRs), which provide a photosynthesis vs. irradiance curve for a given sample. Discrete samples were analyzed using a Heinz-Walz WATER-PAM (Effeltrich, Germany) [73]. The instrument was blanked with 0.2 μm filtered base water using a Cytiva Whatman GD/X syringe filter (part number 6870-2502). Discrete samples were dark-adapted for 30 min, and then the gain was adjusted on the WATER-PAM. Subsequent samples used the same gain setting, with three technical replicates per sample. The first PAM reading provides variable fluorescence, while subsequent measurements are subjected to increasing irradiance to calculate the relative Electron Transport Rate (rETR) response curve, functionally equivalent to a photosynthesis vs. irradiance (PvsE) curve. The data were fit to the four-parameter curve proposed by Platt et al. [74]. The curve fit provides rETR_max_ (μmol photons m^−2^ s^−1^), or the maximum electron transport rate, α, the initial slope of the curve (μmol electrons m^−2^ s^−1^/μmol photons m^−2^ s^−1^), E_k_ (μmol photons m^−2^ s^−1^), the irradiance at half-saturation, and β (μmol electrons m^−2^ s^−1^/μmol photons m^−2^ s^−1^), a photo-inhibition term. rETR is typically reported in relative units.

### 5.8. Pinto Lake Column Experiments

For Pinto Lake water, additional experiments were conducted in 0.5 m (~1 L) and 1.5 m (~20 L) columns to assess the ability of C7X1 to penetrate a vertical column and suppress surface aggregation. Pinto Lake water was added to the columns, allowed to equilibrate for 24 h in an environmental chamber, and then exposed to C7X1 using a spray bottle to simulate typical deployment conditions where the algaecide is sprayed onto the surface of a bloom. One set of experiments (0.5 m columns) also tested the effect of adding the C7X1 at the surface and then immediately mixing the full column, which essentially replicates the 5 mL benchtop testing at scale. The 0.5 m columns were kept in environmental chambers (20 °C) for up to five days with testing at the beginning and end, while the 1.5 m columns, which used Pinto Lake water collected 28 January 2025, were maintained for 4 weeks at 15 °C to match typical field conditions for January, with sampling at 1, 3, 5, and 7 days and then weekly. The columns were sampled at varying depths by inserting a glass tube sealed at the top end so that water could be collected from specific depths without disturbing the vertical structure of the column. Sampling took place by removing and replacing the seal at the top to collect a small volume of water at depth. Replicates (control and treatment) were conducted sequentially for logistical reasons.

### 5.9. Hetereosigma akashiwo Column Experiments

A third set of column experiments was conducted with *H. akashiwo* strain EBL 71. Stock cultures in late exponential growth were diluted into 2 L cell-free media at a final concentration of 39.4 µg L^−1^ chlorophyll and dispensed into four columns, which were held for 24 h in an environmental chamber. Columns were Control (replicates) and treated (replicates) with surface spray and no mixing. Sampling was conducted as described above. Algaecide concentration was reduced to 1000 ppm equivalent for the column volume based on IC_50_ values obtained for the same culture prior to the experiment.

### 5.10. Toxin Testing

For the *Microcystis* and *Pseudo-nitzschia* cultures, samples from the IC_50_ experiments were analyzed at 24 h for release of toxins, while the 0.5 m column experiments with Pinto Lake water were tested at 24 h from the surface and at the bottom of the columns for control and treatment columns. Approximately 1 mL of whole water was collected for total (cellular and dissolved) toxins, and a second 1 mL sample was syringe filtered under low pressure using a 13 mm PVDF syringe filter (Waters Pall Acrodisc, Cortland, NY, USA, part number 4450T). The filtrate was analyzed for dissolved toxin. Domoic acid and microcystin LR, LA, YR, and RR were analyzed on an Agilent 6150 LC/MS with JetSpray Technology and Select Ion Monitoring (Santa Clara, CA, USA) using an external calibration curve with pure standards obtained from NRC Canada (Ottawa, Canada) [11]. Since the primary interest for this analysis was the release of toxins upon exposure to C7X1, concentrations are reported as percent dissolved toxin relative to the control samples, but initial toxin concentrations (µg L^−1^) are reported for the control samples.

## Figures and Tables

**Table 1:** Ingredients in the Anjon AG C7X1 algaecide. (A) indicates active ingredients, (I) indicates inactive ingredients.Not Applicable (N/A) indicates ingredients with no CAS number.

Ingredient Name	CAS	Percent (%)
Peppermint (A)	N/A	0.1
Lemongrass Oil (A)	8007-02-1	1.0
Cinnamon (A)	N/A	2.0
Cottonseed Oil (A)	8001-29-4	2.0
Geranium Oil (A)	8000-46-2	2.0
Thyme (A)	N/A	2.0
Rosemary (A)	N/A	3.0
Clove (A)	N/A	4.0
Garlic (A)	N/A	4.0
Total Active Ingredients:		20.1
Citrus Pectin (I)	9000-69-5	
Citrus Peel Extract (I)	94226-47-4	
Guar Gum (I)	9000-30-0	
Isopropyl Alcohol (I)	67-63-0	
Water (I)	N/A	
Xanthan Gum (I)	11138-66-2	
Total Inactive Ingredients:		79.1

**Table 2:** The concentration of C7X1 at which F_v_/F_m_ is inhibited by 50% (IC_50_) and the standard error for the curve fit (SE) using Equation (2) (concentrations are ppm), and the dose at which F_v_/F_m_ was <0.05 after 24 h.

Algal Strain	IC_50_ (SE)	F_v_/F_m_ < 0.05
*Anabaena flos-aquae*	13,247 (6962)	20,000
*Gloeocapsa alpicola*	2015 (292)	10,000
*Microcystis aeruginosa*	976 (96)	6000
*Chattonella marina*	393 (135)	10,000
*Chattonella subsalsa*	713 (140)	10,000
*Heterosigma akashiwo*	1618 (835)	5000
*Isochrysis galbana*	3456 (571)	10,000
*Pseudo-nitzschia multiseries*	4073 (748)	10,000

**Table 3:** Algal cultures used for testing. UTEX is the University of Texas at Austin culture collection, NCMA is the National Center for Marine Algae and Biota, and EBL is the Environmental Biological Laboratory at Moss Landing Marine Labs, San Jose State University. Standard errors for growth rates are provided in parentheses.

Algal Strain (Class)	Strain/ID	Source	Temperature (°C)	Salinity (PSU)	Growth Rate (d^−1^)
*Anabaena flos-aquae* (Cyanophycae)	B 1444	UTEX	~20	0	0.173 (0.084)
*Gloeocapsa alpicola* (Cyanophycae)	589	UTEX	~20	0	0.172 (0.02)
*Microcystis aeruginosa* (Cyanophycae)	LB 2385	UTEX	~20	0	0.072 (0.002)
*Chattonella marina* (Raphidophyceae)	CCMP 2049	NCMA	20	32	0.229 (0.025)
*Chattonella subsalsa* (Raphidophyceae)	CCMP 2821	NCMA	20	32	0.433 (0.029)
*Heterosigma akashiwo* (Raphidophycae)	EB L71	EBL	15	24	0.192 (0.051)
*Isochrysis galbana* (Coccolithophyceae)	CCMP 1323	NCMA	15	32	0.314 (0.003)
*Pseudo-nitzschia multiseries* (Bacillariophyceae)	EBL 123	EBL	15	32	0.285 (0.028)

## Data Availability

The original contributions presented in this study are included in the article. Further inquiries can be directed to the corresponding author.

## Appendix A

### Field Sampling

Water was collected from Pinto Lake, CA, USA (36.95° N, 121.77° W), as part of this analysis. Pinto Lake is a shallow spring-fed natural lake 8.3 km inland from Monterey Bay and covers 37 surface hectares. Approximately weekly sampling is conducted from the boat dock on the south end of the lake. Relevant environmental data bracketing the collection dates are provided in Table A1.

**Table A1:** Environmental conditions at Pinto Lake. Community Composition uses a relative abundance index: Rare (R): <1%; Present (P): 1–<10%; Common (C): 10–<25%; Abundant (A): 25–<50%.

Date	Temperature (°C)	Chlorophyll (µg L^−1^)	Community Composition
7/31/24	24.3	900.6	A—Dolichospermum, R—Microcystis, Aphanizomenon, Straustrum, Cymbella
8/6/24	24.7	546.9	A—Microcystis, R—Aphanizomenon, Dolichospermum, Straustrum, diatom (Leptocylindrus-like)
8/13/24	24	55.8	A—Microcystis, R—Aphanizomenon, Dolichospermum, Straustrum, Ceratium
8/20/24	24.3	59.6	A—Microcystis, R—Aphanizomenon, Dolichospermum, Oscillatoria, Ceratium
8/27/24	23.7	45.4	A—Microcystis, R—Aphanizomenon, Dolichospermum, Straustrum
9/3/24	23.7	78.3	A—Microcystis, P—Dolichospermum, R—Aphanizomenon, Ceratium
9/17/24	22.1	77.7	A—Microcystis, C—Dolichospermum, R—Aphanizomenon, Ceratium
9/24/24	22.3	85.9	A—Microcystis, P—Dolichospermum, R—Aphanizomenon, Pediastrum
10/1/24	21.9	74.9	A—Microcystis, R—Dolichospermum, Pediastrum
10/15/24	21.4	120.4	A—Microcystis, R—Dolichospermum, Oscillatoria, Ceratium, diatom (Leptocylindrus-like)
10/22/24	18.9	957.1	A—Microcystis, R—Dolichospermum, Aphanizomenon, Oscillatoria, flagellate
11/5/24	16.9	2446.3	A—Microcystis, R—Aphanizomenon, Ceratium
11/12/24	15.9	188.2	A—Microcystis
11/25/24	14.4	174.0	A—Microcystis, R—Ceratium
12/3/24	12.8	1340.3	A—Microcystis, R—Aphanizomenon
12/10/24	11.7	372.4	A—Microcystis, R—Aphanizomenon
12/17/24	12.2	1360.4	A—Microcystis, R—Aphanizomenon
1/7/25	12	12.6	C—Aphanizomenon, Microcystis, R—Ceratium, flagellate
1/14/25	10.6	6.6	C—Aphanizomenon, Microcystis, R—Ceratium, flagellate, tons of sparkles/bacteria
1/21/25	10.3	5.5	C—Aphanizomenon, Microcystis, R—Ceratium
2/5/25	12.9	3.6	A—Microcystis, P—Aphanizomenon, R—flagellate
2/11/25	11.3	2.1	A—Microcystis, R—flagellate
2/18/25	12.5	1.7	A—Microcystis, R—Ceratium
2/25/25	14.8	2.1	C—Microcystis, Ceratium, Coelastrum, tons of sparkles/bacteria
3/4/25	14.3	2.8	C—Microcystis, Ceratium, diatom (Leptocylindrus-like), tons of sparkles/bacteria,
3/18/25	14.5	6.7	A—Microcystis, R—Ceratium
3/25/25	18.5	6.9	C—Microcystis, Aphanizomenon, flagellate, R—Ceratium, Coelastrum, diatom (Leptocylindrus-like)
